# Direct ascending aortic versus peripheral arterial cannulation for type A acute aortic dissection

**DOI:** 10.1186/1749-8090-8-S1-O22

**Published:** 2013-09-11

**Authors:** K Kamiya, K Doi, K Meshii, Y Okada, Y Soga

**Affiliations:** 1 Division of Cardiovascular Surgery, Mie Heart Center, Mie, Japan; 2 Division of Cardiovascular Surgery, Nagahama City Hospital, Shiga, Japan

## Background

The optimal site of arterial cannulation in acute type A aortic dissection (AAD) surgery remains controversial. We retrospectively investigated our experience with ascending aortic (central) cannulation as an alternative to femoral or axillary (peripheral) procedures.

## Methods

We enrolled consecutive 45 patients who underwent type A AAD repair from January 2007 to March 2012. Central cannulation was applied under ultrasound guidance using the Seldinger technique in 33 patients and in 12 patients through peripheral cannulation. After the distal aortic anastomosis, antegrade systemic reperfusion was established with prosthetic side arm. Two groups were compared on the basis of comorbidities, mortality, and complications.

## Results

Preoperative patient characteristics were almost comparable between two groups. Central cannulation was safely performed in all 33 cases. Preoperatively, thirteen patients (28.9%) had shock state with cardiac tamponade, the overall hospital mortality was 15.6%. Intraoperative data including operation time, cardiopulmonary bypass (CPB) time, and selective cerebral perfusion showed significantly shorter in the central group (P<0.05). In particular, the central group had significantly shorter time in central cooling and re-warming during CPB than the peripheral group (P<0.0001). In postoperative morbidities, the central group had shorter mean ventilation time (P=0.03) and the duration of ICU stay (P=0.002). Additionally, the central group experienced significantly fewer acute lung injury (ALI) than the peripheral group (P=0.04).

## Conclusions

Direct central cannulation produced acceptable early surgical outcomes. This procedure especially enabled to establish the rapid cooling and re-warming on CPB, which might contribute to the lower incidence of postoperative ALI than the conventional peripheral access. Our results suggested ascending aorta is a safe alternative cannulation site for the repair of type A AAD.

